# Bullying prevention as a preventive strategy for mental health

**DOI:** 10.1192/j.eurpsy.2023.154

**Published:** 2023-07-19

**Authors:** C. M. Díaz-Caneja

**Affiliations:** Department of Child and Adolescent Psychiatry, Institute of Psychiatry and Mental Health, Hospital General Universitario Gregorio Marañón, IiSGM, CIBERSAM, School of Medicine, Universidad Complutense, Madrid, Spain

## Abstract

**Abstract:**

Bullying constitutes a major public health concern, on account of its high prevalence rates and its association with a wide range of adverse health outcomes across the lifespan, including increased incidence of mental disorders such as depression, anxiety, and psychotic disorders. Previous research suggests effectiveness of school-based programmes in reducing bullying prevalence and improving mental health outcomes in children and adolescents. Despite the fact that some subpopulations such as young people with special educational needs are at increased risk for both bullying victimisation and mental health difficulties, there is little information on the effectiveness of universal school-based programmes in these high-risk populations. We will review available evidence of the effectiveness of school-based anti-bullying interventions as a tool to improve youth mental health, including results from a cluster-randomised clinical trial conducted in 20 publicly funded schools in Madrid to test the effectiveness of a 12-week web-enabled, user-friendly, school-based, preventive programme incorporating universal and targeted components (LINKlusive; ISRCTN15719015) and discuss the potential implications, challenges, and unmet needs of such approaches.

**Disclosure of Interest:**

None Declared

